# Xanthomicrol Activity in Cancer HeLa Cells: Comparison with Other Natural Methoxylated Flavones

**DOI:** 10.3390/molecules28020558

**Published:** 2023-01-05

**Authors:** Mariella Nieddu, Federica Pollastro, Paola Caria, Stefano Salamone, Antonella Rosa

**Affiliations:** 1 Department of Biomedical Sciences, University of Cagliari, 09042 Monserrato, Italy; 2 Department of Pharmaceutical Sciences, University of Eastern Piedmont “Amedeo Avogadro”, 28100 Novara, Italy; 3 PlantaChem Srls, Via Amico Canobio 4/6, 28100 Novara, Italy

**Keywords:** natural flavones, xanthomicrol, cancer cells, cytotoxicity, lipids, cell cycle

## Abstract

The methoxylated flavone xanthomicrol represents an uncommon active phenolic compound identified in herbs/plants with a long application in traditional medicine. It was isolated from a sample of *Achillea erba-rotta* subsp. *moschata* (musk yar-row) flowering tops. Xanthomicrol promising biological properties include antioxidant, anti-inflammatory, antimicrobial, and anticancer activities. This study mainly focused on the evaluation of the xanthomicrol impact on lipid metabolism in cancer HeLa cells, together with the investigation of the treatment-induced changes in cell growth, morphology, and apoptosis. At the dose range of 5–100 μM, xanthomicrol (24 h of incubation) significantly reduced viability and modulated lipid profile in cancer Hela cells. It induced marked changes in the phospholipid/cholesterol ratio, significant decreases in the levels of oleic and palmitic acids, and a marked increase of stearic acid, involving an inhibitory effect on de novo lipogenesis and desaturation in cancer cells. Moreover, marked cell morphological alterations, signs of apoptosis, and cell cycle arrest at the G2/M phase were observed in cancer treated cells. The bioactivity profile of xanthomicrol was compared to that of the anticancer methoxylated flavones eupatilin and artemetin, and structure–activity relationships were underlined.

## 1. Introduction

Among secondary metabolites, flavonoids are a well-known group of polyphenolic compounds characterized by a benzo-γ-pyrone structure and occurring broadly in plants and foods/beverages of natural sources [1,2,3]. The great interest in these compounds is attributable to their various bioactive effects, including antioxidant, antiviral, anti-inflammatory, cardioprotective, antidiabetic, antiaging, and anticancer properties [1,2,4]. Numerous flavonoids exhibit the ability to inhibit cancer cell viability, cancer cell proliferation, angiogenesis, and migration [1,2,4], and dietary flavonoid intake is associated with a reduced risk of different types of cancer, such as gastric, breast, prostate, and colorectal cancers [5]. Several mechanisms of action have been proposed for the anticancer activity of flavonoids, including induction of apoptosis, nuclear factor signaling inhibition, cell cycle arrest induction, interaction with carcinogenic associated enzymes, modulation of ROS-scavenging enzyme activities, proteasome inhibition, differentiation induction, and estrogen receptor binding capacity [1,2,3,4]. In addition, cell lipid metabolism has been suggested as another possible target of natural flavonoids in cancer cells [3,6].

**Figure 1:**
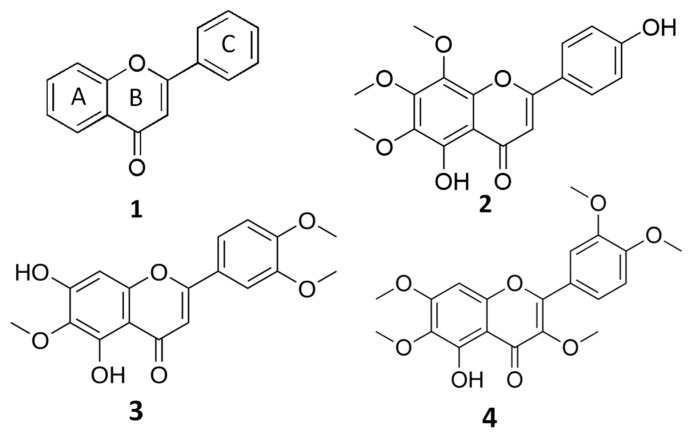
General structure of flavones (**1**) and chemical structures of xanthomicrol (XAN, (**2**)), eupatilin (EUP, (**3**)) and artemetin (ART, (**4**)).

Flavones are structurally characterized by a 2-phenylchromen-4-one backbone (Figure 1), and the different substitution patterns on their basic structures (C6—C3—C6 rings) greatly influence their bioactivity and bioavailability [7,8,9]. A deep correlation between the anticancer properties of flavones and their structure (number and position of hydroxyl and methoxyl groups) has been evidenced [7,8].

The biochemical class of the methoxylated flavones represents uncommon phenolic compounds in plants, characterized by a marked lipophilic behavior due to the alkylation of their hydroxylic groups [3,7,8,9]. Methoxylated flavones have been demonstrated to possess great potential as chemopreventive/chemotherapeutic agents superior to the more common unmethylated flavonoids or polyphenols [7,8,9]. Properties such as molecular weight and lipophilicity, established by the number and type of substituents (hydroxyl, methoxyl, prenyl, and glycosyl groups), are mainly involved in flavonoid bioavailability (absorption, distribution, metabolism, and excretion) [10]. Generally, flavonoids are assumed to penetrate the plasma membrane both by passive diffusion and by transporters [10,11], and flavonoid structural properties as the partition coefficient (log P3), the total number of H-bonds, and topological surface area (TPSA) [12] can reveal how these compounds penetrate the cell membrane [10]. It has been demonstrated that methylation of free phenolic hydroxyl groups on the flavone backbone leads to derivatives not susceptible to glucuronic acid or sulfate conjugation, and greatly improves transport through biological membranes, such as in intestinal absorption, resulting in increased metabolic stability and oral bioavailability [8,9]. Moreover, it is well known that flavonoids can affect cell membrane fluidity and properties, interacting with the lipid membrane surface or inserting themselves into the lipid bilayer [10,13,14]. The flavonoid modulation of membrane biophysical parameters, including modification of the membrane lipid bilayer and protein structures, fluidity, lipid molecules packing, and hydration, depends on their lipophilicity, shape, and location in the bilayer [10,13,14].

The *O*-methylated flavone xanthomicrol (5,4′-dihydroxy-6,7,8-trimethoxyflavone) (XAN, 1) (Figure 1) is a lipophilic compound (the partition coefficient for the octanol–water mixture: log P3 = 2.9, Table 1) [12] presenting two free hydroxyl group in the A and C rings (Figure 1).

XAN is identified as an active compound in herbs/plants with a long application in traditional medicine, such as *Baccharis pentlandii* D.C. [15], *Dracocephalum kotschyi* Boiss [16,17,18,19], *Ocimum gratissimum* L. [20], *Baccharis densiflora* Wedd [21], and *Artemisia campestris* L. [22]. Several studies reported the antioxidant [7], anticancer [7,17,18,19,21,23], antimicrobial [7,22], and anti-inflammatory [7] properties of XAN. The antitumoral activity of XAN has been related to its ability to inhibit cancer cell viability/proliferation [7,17,18,21,23], angiogenesis [16], cancer-related enzymes [19], and to induce apoptosis [17,23] and cell cycle arrest [23].

In this manuscript, for the first time, we investigated the ability of the methoxylated flavone XAN to affect the lipid profile in cancer cells with the aim to provide new insights into its antitumor properties/mechanism. Lipid metabolism is considered a promising anticancer target [24,25], and the antitumoral properties of methoxylated flavones have also been related to their ability to affect cell membrane fluidity and the general lipid membrane organization and structure, inhibiting the expression of enzymes involved in lipid metabolism [3,6]. We previously demonstrated the role of the natural methoxylated flavones eupatilin (5, 7-dihydroxy-3′,4′,6-trimethoxyflavone; EUP) [6] and artemetin (5-hydroxy-3,6,7,3′,4′-pentamethoxyflavone; ART) [3] (Figure 1) as modulatory agents on cancer cell physiology, especially impacting viability, lipid metabolism, cell fluidity, and mitochondrial potential. Changes in lipid components occurring in XAN-treated cancer HeLa cells (24 h of incubation) were explored by monitoring cell phospholipids (PL), free cholesterol (FC), and total fatty acid (FA) composition. The impact on lipid profile was analyzed in XAN-treated cancer cells together with the investigation of the changes occurring in the cell growth, cell morphology, cell cycle, and apoptosis appearance. Finally, the XAN effect on cell viability was tested in 3T3 fibroblasts, a normal cell line previously used to assess the toxicity and biocompatibility of nanoparticles and natural compounds [26]. The bioactivity profile of XAN was compared to that of the anticancer flavones EUP [6], a XAN chemical analog, and ART, characterized by a higher extent of methoxylation in the A and C rings than XAN [3], in the perspective to underline structure–activity relationships.

## 2. Results

### 2.1. Cytotoxic Activity (MTT Assay)

**Figure 2:**
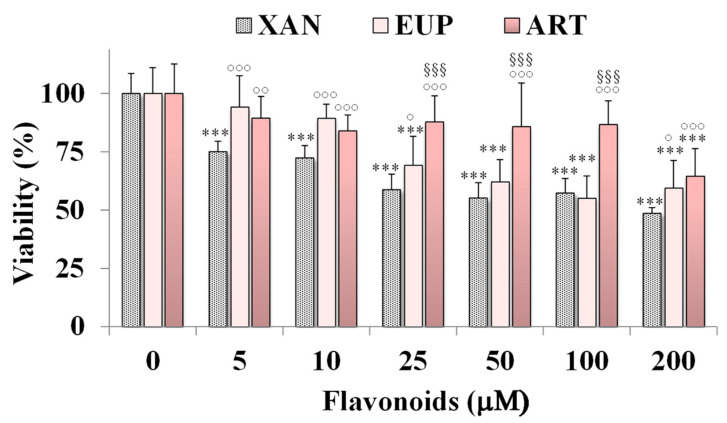
Viability, expressed as % of the control (0), induced by incubation for 24 h with different amounts (5–200 μM) of xanthomicrol (XAN), eupatilin (EUP), and artemetin (ART) in cancer HeLa cells (MTT assay). Three independent experiments are performed, and data are presented as mean and SD (*n* = 15). For each series: *** = *p* < 0.001 versus Ctrl. For each concentration group: °°° = *p* < 0.001, °° = *p* < 0.01, ° = *p* < 0.05 versus XAN-treated cells; ^§§§^ = *p* < 0.001 versus EUP-treated cells. Statistical significance of differences was assessed by One-way ANOVA and Bonferroni post Test.

The cytotoxicity of XAN was investigated on HeLa cells, a cancer cell line previously used to assess the cytotoxic effect of natural flavonoids [3,6]. Figure 2 shows the viability, expressed as % of the control, induced by the 24 h treatment with different amounts (from 5 to 200 μM) of XAN in cancer HeLa cells by the MTT cell viability assay. 

The cytotoxic effect of the reference methoxylated flavonoids EUP [6] and ART [3] was also reported in Figure 2 for comparison. XAN exerted a significant (*p* < 0.001) reduction (25%) in HeLa cell viability, in comparison with control cells, from the dose of 5 μM. A cancer cell growth inhibition of 28–43% was observed at the concentration range of 10–100 μM, while a 51% viability reduction was observed at the highest tested dose (200 μM). The IC_50_ value (the concentration that decreases the cell viability to 50%) of XAN after 24 h incubation in cancer HeLa cells was 182 μM. An analogous treatment with the bioactive flavone analog EUP (log P3 = 2.9, Table 1) [6] induced a lower decrease in cancer HeLa cell viability than XAN in the range 5–25 μM, whereas similar values of toxicity were observed from 50 μM. However, it was not possible to determine the exact IC_50_ value for EUP in HeLa cells at 24 h incubation because this value exceeds the solubility of the compound and the maximum tolerated DMSO percentage (2%) in cell cultures [6]. The lipophilic flavone ART (log P3 = 3.4, Table 1) [3] showed a lower cytotoxic effect than XAN at all tested concentrations, inducing a cancer cell viability reduction of 11–16% at the concentration range of 10–100 μM, and 36% at 200 μM. DMSO, used to dissolve flavonoids, was not toxic in HeLa cancer cells, and at the maximal tested dose (2%), the cell viability was 93%.

**Figure 3:**
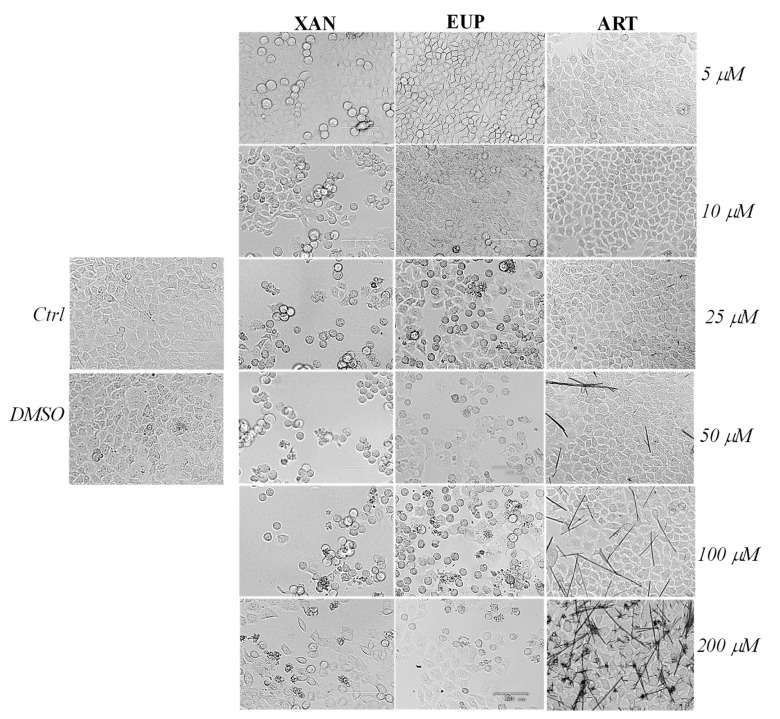
The panel shows representative images of phase contrast of control HeLa cells and cells treated for 24 h with xanthomicrol (XAN), eupatilin (EUP), and artemetin (ART) at 5–200 μM. Bar = 100 μm.

Microscopic observation of HeLa cells treated for 24 h with XAN (Figure 3), before the MTT assay, showed evidence of changes in cell morphologies with respect to control cells from the dose of 5 μM.

Control HeLa cells were small and closely linked to each other (packed), while the XAN treatment induced a remarkable increase in the number of apoptotic cells (characterized by a rounded morphology) in a concentration-dependent manner. Moreover, the occurrence of clear apoptotic bodies and cell debris was observed at the highest XAN concentrations (100 and 200 μM). The microscopic observation of EUP-treated cells evidenced less marked morphology changes and a smaller number of apoptotic (rounded) cells than XAN in the range of 5–25 μM, while a comparable increase in the number of rounded cells was observed from 50 μM. The occurrence of some rounded cells in ART-treated cancer HeLa was evident only at the highest tested doses (100 and 200 μM) moreover, a marked drug precipitation in form of crystals was observed from the dose of 50 μM, which may be responsible for the observed ART cytotoxicity. XAN and the chemical analog EUP shared a similar cytotoxic profile and impact on cell morphology in cancer HeLa cells.

**Figure 4:**
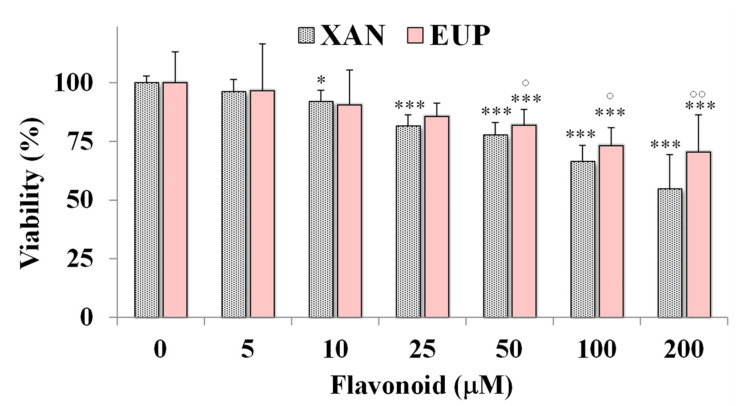
Viability, expressed as % of the control (0), induced by incubation for 24 h with different amounts (5–200 μM) of xanthomicrol (XAN) and eupatilin (EUP) in murine 3T3 fibroblasts (MTT assay). Three independent experiments are performed, and data are presented as mean and SD (*n* = 15). For each series: *** = *p* < 0.001, * = *p* < 0.05 versus respective controls (One-way ANOVA and Bonferroni post Test). For each concentration group: °° = *p* < 0.01, ° = *p* < 0.05 for EUP-treated cells versus XAN-treated cells (Student’s unpaired t-test with Welch’s correction).

Then, the cytotoxic effect of XAN was investigated on 3T3 murine fibroblasts, a normal cell line, and compared to that of EUP. Figure 4 shows the viability, expressed as % of the control, induced by the 24 h treatment with different amounts (5–200 μM) of XAN and EUP in 3T3 fibroblasts by MTT assay.

No marked changes in cell viability, with respect to control cells, were observed in 3T3 fibroblasts treated with XAN at 5 and 10 μM (viability reduction < 10%). A significant viability reduction, compared to controls, ranging from 19 to 46%, was observed for XAN in 3T3 cells from the dose of 25 μM to 200 μM. However, a less marked cytotoxic effect was observed for XAN (Figure 4) in normal fibroblasts with respect to cancer HeLa cells in the amount range of 5–100 μM (*p* < 0.001 at all the tested concentrations versus HeLa cells), indicating more selective toxicity towards malignant cells than normal cells. By a preliminary microscopic observation, no marked effect of XAN on 3T3 cell morphology was observed in the amount range of 5–100 μM. As observed in HeLa cells, EUP showed no cytotoxicity in the range of 5–25 μM and a significantly lower cytotoxic effect than XAN from 50 μM, evidencing selective toxicity versus cancer cells.

### 2.2. Effects on HeLa Cell Cycle Progression

**Figure 5:**
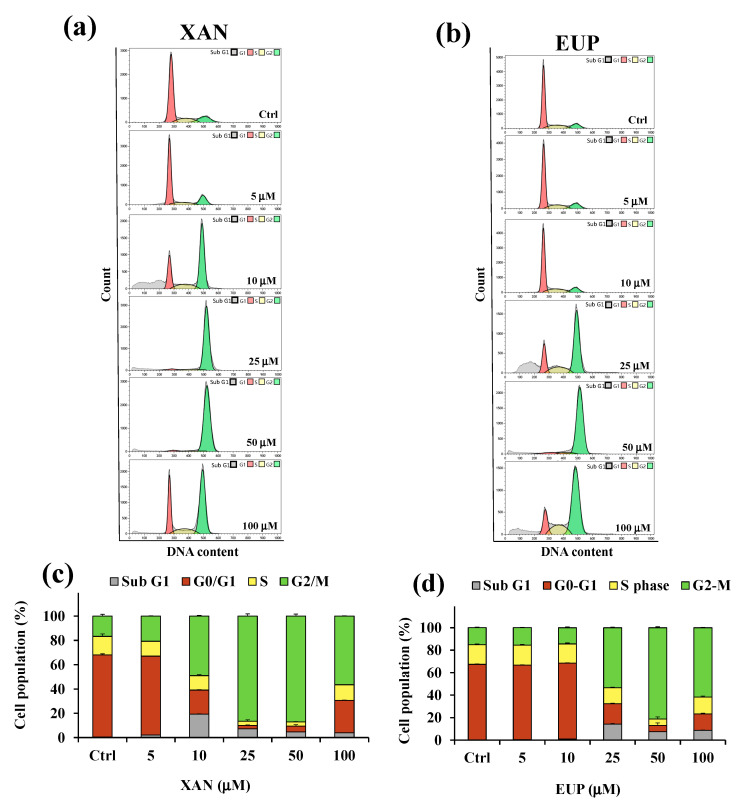
Flow cytometric analysis of the cell cycle distribution determined in control HeLa cells (Ctrl) and cells treated for 24 h with xanthomicrol (XAN) (**a**) and eupatilin (EUP) (**b**) at the concentration range of 5–100 μM. Percentage values of the HeLa cells in cell cycle patterns (sub G1, G0/G1, S, and G2/M phases) determined for XAN (**c**) and EUP (**d**). Three independent experiments are performed, and data are presented as mean and SD (*n* = 3).

Decreased HeLa cell numbers observed in the MTT assay could be explained by either the cell cycle arrest or apoptosis induction. Therefore, the effect of XAN on the cell cycle progression of cancer HeLa cells was determined using the flow cytometry method. Flow cytometric analysis of the cell cycle distribution determined in control HeLa cells and cells treated for 24 h with XAN and EUP at the range concentration 5–100 μM was reported in Figure 5.

Flow cytometry analysis showed that, compared with the control, the cancer HeLa cells treated with XAN (Figure 5a,c) displayed an increase in the percentage of cells at the sub-G1 phase from 5 μM to 100 μM, indicating an increase in the number of apoptotic cells (sub-G1 population) compared to control cells. The exposure of cells to XAN resulted, at the dosage range of 5–50 μM, in a dose-dependent accumulation of the proportion of cells in the G2/M phase, and a clear cell cycle arrest at the G2/M phase was observed at 25 and 50 μM. Interestingly, cancer HeLa cells treated with XAN 100 μM showed a distribution of cell population similar to that observed for 10 μM XAN-treated cells. The treatment with EUP (Figure 5b,d) for 24 h did not affect the distribution of the cancer HeLa cell population with respect to control cells at the dosages of 5 and 10 µM. An increase in the percentage of the sub-G1 population with respect to control cells was observed from 25 µM, coupled to an accumulation of % cells in the G2/M phase, indicative of a cell cycle arrest at this phase. After treatment with 25, 50, and 100 μM EUP, the percentage of cells in the G2/M phase was 53.3%, 81.1%, and 61.6%, respectively, compared to 15.1% in the control cells.

Our results confirmed the cytotoxicity of XAN in cancer HeLa cells, with minor toxic effects in normal cells, and the involvement of the activation of cell apoptosis and cell cycle arrest in the chemopreventive activities of the flavone. For the evaluation of XAN modulatory effect on lipid metabolism in cancer, HeLa cells dosages of 5, 10, and 25 µM were selected, due to their low level of toxicity to normal fibroblasts.

### 2.3. Effect on Cell Lipid Components

**Figure 6:**
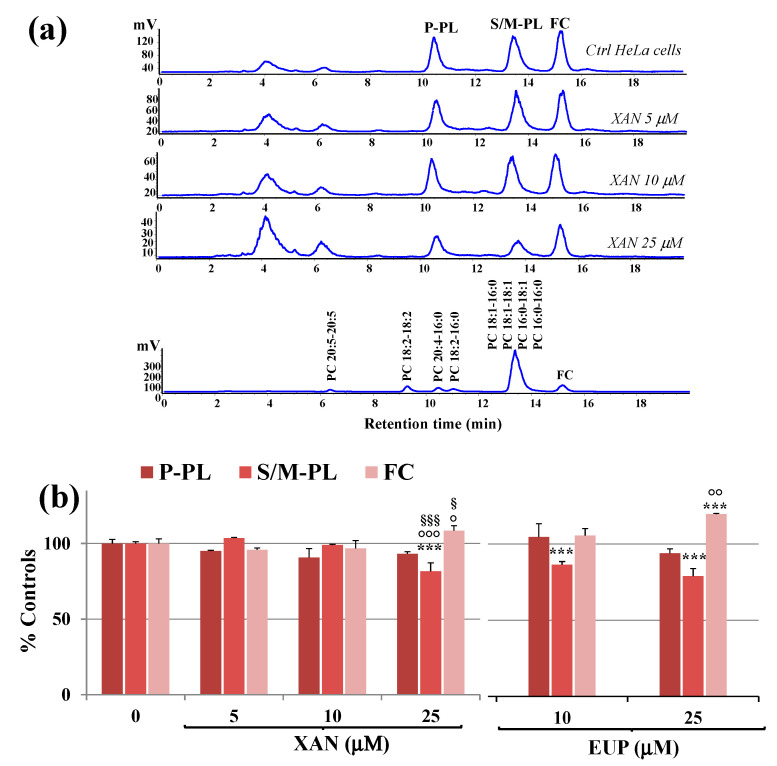
(**a**) Chromatographic profile obtained by HPLC-ELSD analysis at 0.7 mL/min of polyunsaturated phospholipids (P-PL), saturated/monounsaturated phospholipids (S/M–PL), and free cholesterol (FC), measured in control HeLa cells (0) and cells treated for 24 h with different amounts (5, 10 and 25 μM) of xanthomicrol (XAN). A standard mixture of saturated/monounsaturated (mix PL: PC 16:0/16:0, PC 18:1/18:1, PC 16:0/18:1, PC 18:1/16:0) and polyunsaturated phosphatidylcholines (PC 16:0/18:2, PC 16:0/20:4, PC 18:2/18:2; PC 20:5/20:5) were used to assign the chromatographic region for each lipid class. (**b**) Values (% controls) of PL and FC measured in control and HeLa cells treated with XAN (5, 10 and 25 μM) and the reference compound eupatilin (EUP) (10 and 25 μM) [6]. Three independent experiments are performed, and data are presented as mean ± standard deviation (SD) (*n* = 6). For each series: *** = *p* < 0.001 versus Ctrl; °°° = *p* < 0.001, °° = *p* < 0.01, ° = *p* < 0.05 versus cells treated with XAN 5 μM; ^§§§^ = *p* < 0.001, ^§^ = *p* < 0.05 versus cells treated with XAN 10 μM. Statistical significance of differences was assessed by One-way ANOVA and Bonferroni post Test.

XAN was then tested in cancer HeLa cells to assess its effects after 24 h incubation on cancer cell lipid composition, with particular regard to polar lipid classes and total fatty acids (TFA) profile. Total lipids were extracted from cell pellets of control and treated HeLa cells, and aliquots of total lipid extracts were directly analyzed for free cholesterol and phospholipids contents. Figure 6a shows the chromatographic profile of lipid compounds of HeLa control cells and cells treated for 24 h with XAN 5, 10, and 25 μM obtained by HPLC-ELSD analysis (reversed phase mode).

The chromatographic region for each lipid class was assigned by using standard mixtures of saturated, monounsaturated (S/M-PL) and polyunsaturated (P-PL) phosphatidylcholines (PC), and free cholesterol (FC). In our experimental conditions, cell polar lipids (phospholipids, PL; FC) eluted at low retention times. Lipid components were separated based on ECN (=CN−2n, where CN is the number of acyl group carbons and n is the number of double bonds) and lipids containing FA with the same ECN co-eluted. Control HeLa cells showed a polar lipid profile characterized by two main peaks of PL, corresponding to saturated/monounsaturated PL (S/M-PL) and polyunsaturated PL (P-PL), and a peak of FC. Figure 6b shows the values (expressed as % controls) of PL and FC measured in control HeLa cells and cells treated for 24 h with XAN 5, 10, and 25 μM. The values of PL and FC previously measured in EUP-treated cells (10 and 25 μM, at 24 h-incubation) [6] are also reported in Figure 6b for comparison. The treatment with XAN 5 and 10 μM induced some changes, unless not significant, in the lipid profile. The compound markedly affected cancer HeLa cell lipids at the dose of 25 μM, with a significant decrease in the peak areas of S/M-PL (*p* < 0.001 versus untreated cells) and an increase in the % of FC (*p* < 0.001), together with a slight decrease in % P-PL. The chemical analog EUP, at 10 and 25 μM, modulated lipid profile in the same way as XAN, however, this effect was more marked at both the tested concentrations. 

**Figure 7:**
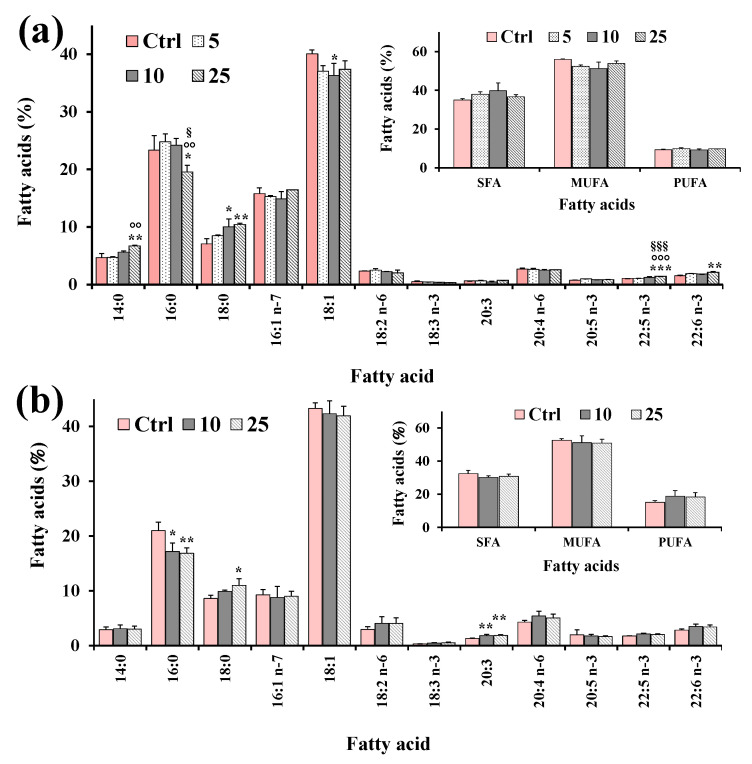
Values (expressed as % of total fatty acids) of the main fatty acids, total saturated (SFA), monounsaturated (MUFA) and polyunsaturated (PUFA) fatty acids measured in control HeLa cells (Ctrl) and cells treated for 24 h with XAN (5, 10 and 25 μM) (**a**) and the reference compound eupatilin (EUP) (10 and 25 μM) [6] (**b**). Three independent experiments and two replicates for each condition are performed, and data are presented as mean ± SD (*n* = 6). *** = *p* < 0.001, ** = *p* < 0.01, * = *p* < 0.05 versus Ctrl; °°° = *p* < 0.001, °° = *p* < 0.01 versus cells treated with XAN 5 μM; ^§§§^ = *p* < 0.001, ^§^ = *p* < 0.05 versus cells treated with XAN 10 μM. Statistical significance of differences was assessed by One-way ANOVA and Bonferroni post Test.

Aliquots of dried lipid extracts, obtained from cell pellets of control and treated HeLa cells, were subjected to mild saponification and then saponifiable fractions were subjected to the analysis of the cell total FA profile. Figure 7 shows the values (expressed as % of total FA) of the main FA, total saturated (SFA), monounsaturated (MUFA), and polyunsaturated (PUFA) measured in control HeLa cells and cells treated for 24 h with XAN (5, 10 and 25 μM) (Figure 7a) and the reference compound eupatilin (EUP) (10 and 25 μM) [6] (Figure 7b).

FA composition of HeLa control cells was characterized by a high level of 18:1 isomers (95.6 ± 7.1 μg/plate, 40% of TFA; mainly 18:1 n-9), palmitic acid 16:0 (55.5 ± 1.0 μg/plate, 23%), palmitoleic acid 16:1 n-7 (37.8 ± 5.8 μg/plate, 16%), stearic acid 18:0 (16.9 ± 3.9 μg/plate, 7%), and while arachidonic acid 20:4 n-6 (6.32 ± 1.1 μg/plate, 3%) represented the most abundant FA among PUFA. The 24 h incubation of cancer HeLa cells with XAN (Figure 7a) induced changes in the FA profile with respect to control cells from the lowest tested dose (5 μM). Interestingly, the HeLa cell treatment with XAN induced a decrease in the % cell amount of 18:1 n-9, coupled with a dose-dependent increase in the 18:0 level. A marked significant reduction (17%, *p* < 0.001 versus control cells) in the levels of 16:0 was observed at XAN 50 μM. Cells treated with DMSO, used to dissolve the compound, showed the same FA profile as untreated cells. The reference compound EUP (10 and 25 μM, at 24-h incubation) (Figure 7b) induced changes in the FA profile, with respect to untreated cells, similar to those observed with XAN, with a reduction in the % levels of 16:0 and 18:1 n-9, together with an increase in the level of 18:0. 

**Figure 8:**
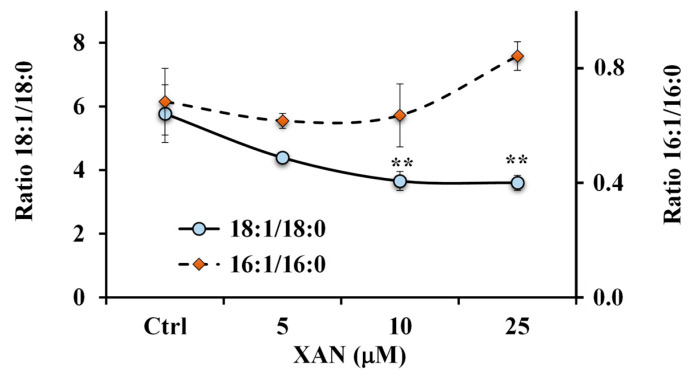
Values of the ratios 18:1 n-9/18:0 and 16:1 n-7/16:0 measured in control HeLa cells and cells treated for 24 h with different amounts (5, 10 and 25 μM) of xanthomicrol (XAN). ** = *p* < 0.01 versus Ctrl. Statistical significance of differences was assessed by One-way ANOVA and Bonferroni post Test.

A marked reduction of the 18:1 n-9/18:0 ratio value (Figure 8) was observed in XAN-treated cells at all tested concentrations with respect to control cells (characterized by the value of 5.8 ± 0.9), and values of 3.7 ± 0.3 (*p* < 0.01) and 3.6 ± 0.2 (*p* < 0.01) were determined in 10 μM and 25 μM-XAN treated cells, respectively. 

Otherwise, an increase, unless not significant, in the 16:1 n-7/16:0 ratio value (Figure 8) was observed in cancer HeLa cells treated with XAN at the dose of 25 μM. 

Control HeLa showed a total FA (TFA)/FC ratio value of 5.5 ± 0.6. The 24 h incubation with XAN induced in treated cells a reduction in the TFA/FC ratio, with a value of 2.6 ± 0.6 (*p* < 0.05 versus untreated cells) at XAN 25 μM, due to a modulation of the total cancer cell lipid profile.

## 3. Discussion

Lipids (phosphoacylglycerols, triacylglycerols, sphingolipids, sterols, and cholesteryl esters) and their main building block fatty acids (FA), have structural, energy, signaling, and immunoregulatory functions, regulating several complex biochemical processes [26,27]. Deregulation of lipid and FA metabolism is one of the most important metabolic hallmarks of cancer cells [24]. Cancer cells show an increased de novo FA biosynthesis and exogenous FA uptake that sustain their rapid proliferative rate and provide an essential energy source during metabolic stress conditions [24,28,29,30]. Higher levels of MUFA, mainly oleic and palmitoleic acids, and increased amounts of palmitic acid 16:0, as the main product of de novo lipogenesis, have been measured in cancer cells and tissues [30]. Targeting altered lipid metabolic pathways (such as fatty acid biosynthesis and desaturation, phospholipids and cholesterol metabolism, and lipid droplet synthesis) is a promising therapeutic strategy for human cancer [24]. Many anticancer compounds are inhibitors of fatty acid synthase (FAS), a multi-enzyme that catalyzes de novo synthesis of 16:0 [27,29], and stearoyl-CoA desaturase (SCD), an enzyme that inserts a double bond in the Δ^9^ position of saturated FA (SFA) to generate MUFA [29]. Recently we substantiated cell lipid metabolism as another possible target of the dietary natural methoxylated flavones EUP and ART in cancer cells [3,6]. The treatment of cancer cells with both phenolic compounds induced marked changes in the phospholipid/cholesterol ratio, significant decreases in the levels of oleic and palmitic acids, and a marked increase of stearic acid, maybe involving an inhibitory effect on de novo lipogenesis and desaturation [3,6].

XAN is a trimethoxylated hydroxyflavone characterized by a certain hydrophobicity, as indicated by its structural characteristics such as the log P3 value, the number of H-bonds formed, and the topological polar surface area (TPSA), as given in Table 1 [12]. XAN has been shown to exert antitumor activity (in vitro and in vivo), which has been associated with its ability to modulate a variety of molecular targets and signaling pathways in different cancer cell types, inducing cytotoxic [7,17,18,21,23,31,32] and antiproliferative effects [7,17,18,21,23], apoptosis [17,23,32], and cell circle arrest [23,32]. We studied, for the first time, the impact of this natural dietary compound on cell lipids in HeLa cells, a cancer cell line derived from a human cervical epithelioid carcinoma, widely used as a model for oncological studies [3,6]. Changes in lipid profile were analyzed in XAN-treated HeLa cancer cells together with the investigation of the effect on cell viability, cell cycle progression, and apoptosis.

At first, a set of experiments was performed to assess XAN cytotoxicity in HeLa cells after 24 h incubation. XAN, like the analog anticancer flavonoid EUP [6,33,34], significantly affected viability in HeLa cells, evidencing lower cytotoxicity versus 3T3 normal fibroblasts. XAN was more potent than EUP at the lowest tested doses but showed a similar cytotoxic profile from 50 μM. Whereas, the more lipophilic pentamethoxylated flavone ART showed a low toxic effect at these experimental conditions due to its low solubility in cell medium [3]. We previously demonstrated the viability reduction effect of ART in cancer HeLa cells at low doses after 72 h of incubation [3]. Previous studies showed the ability of XAN to inhibit the growth of several cancer cell lines (4T1, HL60, AGS, HT29, K562, Saos-2, A2780, B16F10, HCT116, and WEHI-164), substantiating selective toxicity versus malignant cells [17,18,23,31,32]. A certain cytotoxic effect of XAN was also observed in normal human fetal foreskin fibroblasts (HFFF-P16) however, the compound showed a high selectivity towards malignant cells than the well-known anti-tumor compound doxorubicin [17,18].

Microscopic observation displayed marked changes in the cell morphology of XAN-treated HeLa cells (reduced cell density as well as round and granulated appearance, membrane blebbing) after 24 h of incubation, highlighting evident signs of an apoptotic process. The microscopic observation of cells 24 h incubated with the chemical analog EUP evidenced similar morphological changes, with an increase in the number of rounded cells, unless at low doses, morphological alterations were more marked in XAN-treated HeLa cells than in EUP-treated cells. Several studies demonstrated the EUP capacity to induce apoptosis, mitochondrial membrane depolarization, and abnormal mitosis with multi-nucleation (mitotic catastrophe) on cancer cell lines [6,33,34]. We previously demonstrated, by flow cytometry, immunofluorescence, and fluorescence, the ability of EUP 10, 25, and 50 μM to induce apoptosis (round reduced volume cells, membrane blebbing, and formation of apoptotic bodies) in cancer HeLa cells after 24 h of incubation [6]. Moreover, previous studies demonstrated that XAN, after 24 h incubation, can induce apoptosis in breast cancer cells [23] and human colon cancer HCT116 cells [32].

Then, the effect of XAN on the cancer HeLa cell cycle progression was determined using the flow cytometry method and compared to that of EUP. Analysis of the cell cycle showed that XAN induced, after 24 h incubation, cell cycle arrest at the G2/M phase in cancer HeLa cells. XAN at 15 and 21 µM significantly increased the accumulation of human colon cancer HCT116 cells in the G2/M phase, while 21 µM of XAN also led to cell cycle arrest at G1/G0 phase [32]. Another paper reported that the IC_50_ concentration of XAN (35 μg/mL) induced G1-arrest after 24 h of incubation in the breast cancer cells [23]. The flavone EUP, incubated for 24 h in cancer HeLa cells for comparison, triggered cell cycle arrest at the G2/M phase in the same way as XAN. Accordingly, previous research showed the ability of EUP to inhibit the growth of human endometrial cancer Hec1A cells via G2/M phase cell cycle arrest in a dose- and time-dependent manner [34]. Moreover, a significant accumulation of the tsFT210 cells at the G2/M phase was also observed after treatment for 17 h with ART [35]. Moreover, the increase in the percentage of XAN-treated cancer HeLa cells at the sub-G1 phase, compared to controls, was diagnostic of an increased number of apoptotic cells (sub-G1 population), as previously reported [36]. 

Then, the XAN modulatory effect on lipid metabolism was explored in cancer HeLa cells. Many anticancer flavonoids (among others, luteolin and quercetin) are inhibitors of the enzyme FAS [37]. Moreover, flavonoids like the tetrahydroxyflavone kuwanone G [38] showed potential inhibition of SCD, the enzyme that inserts a double bond in the Δ^9^ position of 16:0 and 18:0 to generate 16:1 n-7 and 18:1 n-9 [29,39,40]. The predominant polar lipid class in HeLa cell membranes are phosphatidylcholines (PC) (53%), and PC 16:0/18:1 and PC18:1/18:1 are the dominant ones [41]. In our experimental conditions, palmitic acid and oleic acid represented the most abundant FA measured in control cancer HeLa cells. Our results showed that XAN significantly modulated FA and PL profiles in HeLa cells, with a marked reduction in the levels of 18:1 n-9 and 16:0, a decrease in the amount of S/M-PL coupled with an increase in the FC percentage value. Starting from all these considerations, the observed decrease in the S/M-PL level observed in XAN-treated cells was principally due to the reduction of PL containing 16:0 and 18:1 n-9. Moreover, the reduction of 16:0 induced by XAN in cancer HeLa cells was compatible with a potential inhibition of the enzyme FAS [3,6]. In addition, the reduction of 18:1 n-9 observed in XAN-treated cells may be based on the inhibition of SCD, confirmed by the marked decrease of the 18:1 n-9/18:0 ratio value. Similar modifications in the FA composition (reduction of16:0 and 18:1 n-9 and accumulation of 18:0) and PL (decrease in the % of S/M-PL) was previously observed for the analog flavone EUP in cancer HeLa cells in the range dose from 10 to 50 μM after 24 h incubation [6].

XAN showed the ability to modulate the lipid profile in cancer HeLa cells after 24 h of incubation, maybe reducing lipogenesis and affecting FA desaturation and the biosynthesis of PL. Cancer cells, in contrast to normal cells, depend on lipid synthesis pathways for survival [27,28]. Lipid synthesis is essential for cancer cell growth because the high proliferation of cancer cells requires large amounts of lipids as building blocks for biological membranes and metabolic fuels through oxidation in mitochondria [24,27,28]. Therefore, in cancer cells, abrogation of lipid synthesis through inhibition of lipogenic enzymes, such as the FASN, has a strong impact on general metabolism, resulting in decreased cell growth/proliferation and increased apoptosis of cancer cells [27,28]. An elevated synthesis of MUFA, mainly oleic acids 18:1 n-9, has been measured in cancer cells [29,30]. Oleic acid, the main product of SCD, is essential for the generation of complex lipids such as phospholipids, triglycerides, and cholesterol esters [29]. Among them, phospholipids, together with cholesterol, represent the main building blocks for biological membranes [24]. In cancer cell lines, de novo synthesis of MUFA is often required to generate membranes and maintain membrane fluidity, essential to cell proliferation [42]. Therefore, the 18:1 n-9 depletion due to SCD inhibition influences the phospholipid composition of cellular membranes, subsequently affecting membrane properties [39,40,42]. In cancer cells, changes in lipid components severely affect functional and biophysical properties of cell membranes, inducing changes in membrane structure, fluidity, and organization, perturbing membrane lipid raft and protein dynamics [29,37,39]. It is well-known that the maintenance of physiological cell membrane fluidity is necessary for proper membrane function, cell viability, normal cell growth, and division [42]. It is well demonstrated that the lipid modifications occurring in cancer cells are associated with the activation of cell signaling pathways involved in proliferation and tumorigenesis that are initially activated in the membrane [24,25]. Therefore, the XAN-induced changes in cancer HeLa lipid profile could probably trigger alterations in the general lipid membrane organization and structure, severely altering membrane fluidity, and modulating the interaction of pivotal signaling proteins with membranes [25]. The signaling pathways regulated by these proteins could be modified accordingly, resulting in decreased cell growth/proliferation, induction of apoptotic pathways, and cell cycle arrest. 

There is growing evidence that the alteration of membrane fluidity underlies the apoptotic activity of many anticancer agents [25,43]. It has been proved that flavonoids possess the ability to localize either in the hydrophobic core of the membrane lipid bilayer or at the cell lipid membrane surface, leading to corresponding alterations in the membrane fluidity or rigidity in relation to their chemical properties and hydrophobicity [10,13,14,44]. The partition coefficient (log P) provides information on the hydrophobicity of a molecule; in general, if the value of log P is greater than 1, the molecule is considered to possess a hydrophobic nature [3,44]. For flavonoids, the log P value has been correlated with the extent of their interaction with biological membranes [10,44]. The membrane rigidifying effect of dietary flavonoids is one of the key factors for their anti-cancer activity, and this phenomenon exhibits a dose dependency [44]. Furthermore, the membrane interactions and localization of flavonoids are fundamental in altering membrane-mediated cell signaling pathways [3,44]. The influence of flavonoids on the physical properties of the lipid bilayer may control the arrangement of membrane proteins and the formation of functional complexes responsible for cell signal transduction and the regulation of the metabolism [13]. It is well known that cancer cells rearrange lipid composition/organization to avoid apoptosis and resist anticancer drugs [3]. Taking into consideration the results of this study and previous literature data [6], the biological profile of XAN appeared quite similar to that of EUP. The two flavones share the same structural/chemical properties, such as lipophilicity (identical log P3 = 2.9), the total number of H-bonds, and TPSA [12]. These properties could similarly influence their interaction with the lipid membrane or their location in the bilayer division [10,13] and the interactions with enzymes [8,9,43]. Specific structure–activity relationships for the anticancer activity of flavonoids have been well-established, such as the essential role of the C_2_=C_3_ double bond for strong tumor inhibition and the *O*-methylation that contributes to increased biological activity, which is often associated with ring A polymethoxylation [45]. We presented evidence that XAN, like EUP, induced cytotoxicity, apoptosis, and cell cycle arrest, affecting lipid profile in cancer HeLa cells. However, in our study, some dose-dependent differences were observed between the two methoxylated flavones. The 24 h incubation of HeLa cells with XAN 10 μM induced a marked reduction of viability (28%), a high level of cells with apoptotic morphology, and accumulation of the proportion of cells in the G2/M phase, with slight changes in PL and FA profile. The treatment of HeLa cells with EUP 10 μM induced a slight reduction of viability (11%), marked changes in PL and FA profile, with low levels of apoptotic cells and no effect on the cell cycle.

Thus, XAN, similarly to EUP, significantly affected the metabolic functions of cancer HeLa cells, acting simultaneously through different mechanisms. The lipophilic nature of XAN could allow its interaction with the lipid membrane and the inhibition of enzymes involved in lipid metabolism, which resulted in decreased cell growth/proliferation, increased apoptosis, and cell cycle arrest in cancer HeLa cells. Alternatively, the direct ability of this flavone to affect cell membrane fluidity could probably induce the change in the lipid composition as an adaptive strategy of cancer HeLa cells to modify their membrane fluidity in the presence of an antitumor agent. However, the exact sequence of action of this bioactive compound remained very difficult to assess. Results obtained in HeLa cells for the first time substantiated the modulation of cellular lipid metabolism as another possible chemopreventive mechanism of action of XAN in cancer cells, together with the reduction of viability and the activation of cell apoptosis and cell cycle arrest. Further studies are required to explore the XAN interaction with membranes, and its ability to exert therapeutic effects by modulating the properties of cancer cell membranes.

## 4. Materials and Methods

### 4.1. Chemicals and Reagents

Cholesterol, standards of fatty acids, 1,2-dipalmitoyl-sn-glycero-3-phosphocholine (PC 16:0/16:0), 1,2-dioleoyl-sn-glycero-3-phosphocholine (PC 18:1/18:1), 1-palmitoyl-2-oleoyl-sn-glycero-3-phosphocholine (PC16:0/18:1), 1-oleoyl-2-palmitoyl-sn-glycero-3-phosphocholine (PC 18:1/16:0), 2-linoleoyl-1-palmitoyl-sn-glycero-3-phosphocholine (PC 16:0/18:2), 2-arachidonoyl-1-palmitoyl-sn-glycero-3-phosphocholine (PC 16:0/20:4), 1,2-dilinoleoyl-sn-glycero-3-phosphocholine (PC 18:2/18:2), 1,2-dieicosapentaenoyl-sn-glycero-3-phosphocholine (PC 20:5/20:5), 3-(4,5-dimethylthiazol-2-yl)-2,5-diphenyltetrazolium bromide (MTT), and all solvents used (purity ≥99.9%) were obtained from Sigma-Aldrich (Milan, Italy). Cell culture materials were purchased from Invitrogen (Milan, Italy). FxCycle PI/RNase Staining Solution was obtained by Thermo Fisher Scientific (Waltham, MA, USA). All the chemicals used in this study were of analytical grade. The reference compound EUP and ART (with 98% purity) were isolated from the Swiss chemotype of *Artemisia umbelliformis* Lam. (Asteraceae) and from aerial part of *Artemisia absinthium*, respectively, according to the literature [3,6]. 

### 4.2. General Experimental Procedures

Phytochemistry analysis. ^1^H 400 MHz and ^13^C 100 MHz NMR spectra were measured with Bruker 400 spectrometer (Bruker^®^, Billerica, MA, USA). Chemical shifts were referenced to the residual solvent signal (C_3_D_6_O: δ_H_ = 2.05, δ_C_ = 206.7, 29.9 and CDCl_3_: δ_H_ = 7.25, δ_C_ = 77.0, CD_3_OD: δ_H_ = 3.31, δ_C_ = 49.00). Silica gel 60 (70−230 mesh), RP C-18 silica gel, and Celite^®^ 545 particle size 0.02–0.1 mm, pH 10 (100 g/L, H_2_O, 20 °C), used for low-pressure chromatography and vacuum chromatography was purchased from Macherey-Nagel (Düren, Germany). Purifications were monitored by TLC on Merck 60 F254 (0.25 mm) plates, visualized by staining with 5% H_2_SO_4_ in EtOH and heating. Chemical reagents and solvents were from Aldrich (Darmstadt, Germany) and were used without any further purification unless stated otherwise. Flash chromatography Isolera One with DAD (Uppsala, Sweden), HPLC JASCO Hichrom, 250 × 25 mm, silica UV−vis detector-2075 plus (Oklahoma, Japan).

### 4.3. Xanthomicrol (XAN)

XAN was isolated from a sample of *Achillea erba-rotta* subsp. *moschata* (Wulfen) I.Richardson (musk yarrow) flowering tops collected in the territory of the province of Trento harvested at the full blooming stage. A voucher specimen of the plant (AM-2015) is stored at the Novara laboratories, Novara, Italy. 

Flowering tops (500 g) were extracted with acetone (2 × 5 L) in a vertical percolator at room temperature, affording 24.60 g (4.90%) of dark green syrup. This latter was dissolved at 45 °C in the minimal amount of MeOH and layered at the surface of a cake of RP C-18 silica gel (75 g, ratio extract/RP C-18 1:3) packed with MeOH on a sintered funnel (9 × 15 cm) with a side arm for vacuum. Elution with MeOH (100 mL) gave 18 g of a purified depigmented fraction. The latter was fractionated by low-pressure chromatography (LPC) on silica gel (450 g, petroleum ether−EtOAc gradient from 90:10 to 20:80) to afford four fractions (I, II, III, IV). Fraction I was crystallized with diethyl ether to provide 224 mg of XAN (0.045%) as a yellow powder (98% purity) identified with ^1^H NMR (Figure S1 in the Supplementary Materials) according to data present in literature [46].

### 4.4. Cell Cultures

HeLa cell line, derived from a human cervical epithelioid carcinoma, and mouse 3T3 fibroblasts were obtained from the American Type Culture Collection (ATCC, Rockville, MD, USA). Cells were grown in Dulbecco’s modified Eagle’s medium (DMEM) with high glucose, supplemented with 2 mM L-glutamine, penicillin (100 units/mL)–streptomycin (100 μg/mL), and fetal calf serum (FCS) (10% *v*/*v*), at 37 °C in a 5% CO_2_ incubator. Subcultures of HeLa and 3T3 cells were grown in T-75 culture flasks and passaged with a trypsin-EDTA solution. 

### 4.5. Cytotoxic Activity: MTT Assay

The cytotoxic effect of XAN, EUP, and ART was evaluated in cancer HeLa cells and normal 3T3 fibroblasts by the 3-(4,5-dimethylthiazol-2-yl)-2,5-diphenyltetrazolium bromide (MTT) (Sigma-Aldrich, Milan, Italy) colorimetric assay [3,6]. Cells were seeded in 96-well plates at a density of 3×10^4^ cells/mL (HeLa cells) and 3×10^5^ cells/mL (3T3 fibroblasts) in 100 mL of complete culture medium and cultured for 48 h. Cells were subsequently incubated (24 h) with various concentrations (5–200 μM) of XAN and EUP (from solutions in dimethyl sulfoxide, DMSO) in a fresh medium (treated cells). Treated cells were compared for viability to control cells (non-treated) and cells (vehicle-treated cells) incubated for 24 h with an equivalent volume of DMSO (maximal final concentration, 2%). After incubation, cells were subjected to the MTT viability test, as reported [47]. Color development (absorbance proportional to the number of viable cells) was measured at 570 nm with an Infinite 200 auto microplate reader (Infinite 200, Tecan, Austria), and results were expressed as a percentage of cell viability in comparison with control cells. 

Preliminary evaluation of the cancer HeLa cell morphology after 24 h of incubation with various amounts (5–200 μM) of XAN and the reference compounds EUP and ART was performed by microscopic analysis with a ZOE™ Fluorescent Cell Imager (Bio-Rad Laboratories, Inc., Hercules, CA, USA).

### 4.6. Cell Cycle Analysis of HeLa Cells by Flow Cytometry

The effect of XAN and EUP on the DNA content distribution in cancer HeLa cells was evaluated by flow cytometry analysis. Measurement of the DNA content allows the study of cell populations in various phases of the cell cycle as well as the analysis of DNA ploidy. HeLa cells were seeded in 12-well plates at the density of 4 × 10^4^ cells/mL and cultured for 48 h. Cells were then treated for 24 h with different concentrations (5, 10, and 25 μM) of XAN and EUP (from DMSO solutions). Cells were washed once with PBS, detached, and fixed in 500 μL of ethanol for 2 h. Then, cells were centrifuged, washed, and incubated for 30 min (at room temperature in the dark) with 500 μL of FxCycle PI/RNase Staining Solution according to the manufacturer’s instruction. Stained cells were then analyzed by flow cytometry, measuring the fluorescence emission at 530 nm using a 488 nm excitation laser using a MoFlo Astrios EQ Cell Sorter (Beckman Coulter, Brea, CA, USA). The cell cycle was analyzed using Kaluza Analysis Software (Miami, FL, USA).

### 4.7. Lipid Profile Modulation in Cancer HeLa Cells

Cancer HeLa cells were seeded in T-75 culture flasks (at a density of 3 × 10^5^ cells/10 mL of complete culture medium) and cultured for 48 h. Cells were subsequently incubated for 24 h with XAN (5, 10, and 25 μM, from DMSO solutions) in a complete culture medium (treated cells). Control cells (non-treated cells) and vehicle-treated cells (24 h incubation with 0.25% of DMSO) were also prepared. After different treatments, cells were washed with PBS to remove dead cells, scraped, and centrifuged (at 2000 rpm at 4 °C for 10 min). Hela cell pellets, separated from the supernatants, were subsequently used for the extraction of lipid compounds.

### 4.8. Cell Lipid Extraction and Analysis

Total lipids were extracted from HeLa cell pellets with the chloroform/MeOH/water 2:1:1 mixture (6 mL) as previously reported [43,47]. Dried aliquots of the chloroform fractions after cell pellet extraction, dissolved in MeOH, were injected into an Agilent Technologies 1100 HPLC system equipped with a DAD and an Agilent Technologies Infinity 1260 evaporative light scattering detector (ELSD), for the direct analysis of free cholesterol (FC) and phospholipids (PL) [43]. PL (ELSD detection) and FC (DAD detection at 203 nm) were determined with an Inertsil ODS-2 column (Superchrom, Milan, Italy) and MeOH as mobile phase at a flow rate of 0.7 mL/min. Another aliquot of dried chloroform fractions, dissolved in EtOH, was subjected to mild saponification [43] for the analysis of cell FA as reported [43]. Analyses of unsaturated (DAD detection, 200 nm) and saturated (ELSD detection) FA, obtained from cell lipid saponification, were carried out with a mobile phase of acetonitrile/water/acetic acid (75/25/0.12, *v*/*v*/*v*), at a flow rate of 2.3 mL/min. Collected data were analyzed using the Agilent OpenLAB Chromatography data system, as previously described [43]. Calibration curves of FA were constructed using standards and were found to be linear (DAD) and quadratic (ELSD) (correlation coefficients >0.995) [43].

### 4.9. Statistical Analyses

Evaluation of the statistical significance of differences was performed using Graph Pad INSTAT software (GraphPad Software, San Diego, CA, USA). Results were expressed as mean ± standard deviation (SD), and statistically significant differences were evaluated with *p* < 0.05 as a minimal level of significance. Multiple comparisons of means groups were assessed by one–way analysis of variance (One–way ANOVA) followed by the Bonferroni Multiple Comparisons Test to substantiate statistical differences between groups. Student’s unpaired *t*-test with Welch’s correction, which does not require the assumption of equal variance between populations, was used to compare the means of two groups. 

## 5. Conclusions

Lipid metabolism is considered a promising anticancer target, and several antitumor drugs act through the membranes by changing the general lipid membrane organization and structure. In cancer cells, changes in membrane lipid components severely alter membrane fluidity and protein dynamics, perturbing membrane lipid rafts, and inducing apoptotic pathways, eventually resulting in cell death. Natural methoxylated flavones are considered potential cancer chemopreventive agents. The antitumoral activity of the lipophilic trimethoxyflavone XAN has been related to its ability to inhibit cancer cell viability, cancer-related enzymes and to induce apoptosis and cell cycle arrest. This study, for the first time, highlighted the XAN ability to affect lipid components in cancer HeLa cells after 24 h of incubation, maybe reducing lipogenesis and modifying FA desaturation and the biosynthesis of PL. Our results substantiated the modulation of cellular lipid metabolism as another possible chemopreventive mechanism of action of XAN in cancer cells, together with the reduction of viability and the activation of cell apoptosis and cell cycle arrest. Further studies on the XAN modulatory effect on lipid components in other cancer and normal cell lines and animal models will be necessary to elucidate the sequence of actions in targeting lipid metabolism.

## Figures and Tables

**Table 1:** Properties of flavones computed from chemical structure [12].

Flavone	Log P3	Number of H-Bonds ^1^	Topological Polar Surface Area ^2^
Artemetin	3.4	9	92.7
Eupatilin	2.9	9	94.4
Xanthomicrol	2.9	9	94.4

^1^ Log P3: partition coefficient for an octanol–water mixture, computed by XLogP3 3.0. ^2^ Computed by Cactvs 3.4.8.18.

## Data Availability

The datasets generated and analyzed during the current study are available from the corresponding author upon reasonable request.

## Supplementary Materials

The following supporting information can be downloaded at: https://www.mdpi.com/article/10.3390/molecules28020558/s1, Figure S1: ^1^H NMR (400 MHz) of xanthomicrol (XAN) in CDCl_3_.

## Sample Availability

Samples of the compounds are not available from the authors.
