# Supplementary material for: Xanthomicrol Activity in Cancer HeLa Cells: Comparison with Other Natural Methoxylated Flavones
Source: Molecules. 2023 Jan 5;28(2):558. doi: 10.3390/molecules28020558 (PMC9864045; doi:10.3390/molecules28020558)
Supplement: Supplementary file 1 [file molecules-28-00558-s001.zip › molecules-2111349-supplementary.pdf]

Article

Xanthomicrol Activity in Cancer HeLa Cells: Comparison with Other Natural Methoxylated Flavones

Mariella Nieddu [1], Federica Pollastro [2,3], Paola Caria [1], Stefano Salamone [2] and Antonella Rosa [1,*]

[1] Department of Biomedical Sciences, University of Cagliari, 09042 Monserrato, Italy
[2] Department of Pharmaceutical Sciences, University of Eastern Piedmont "Amedeo Avogadro", 28100 Novara, Italy
[3] PlantaChem Srls, via Amico Canobio 4/6, 28100 Novara, Italy
* Correspondence: anrosa@unica.it; Tel.: +39-070-675-4124

Table of Contents

Figure S1: ^{1}H NMR (400 MHz) of xanthomicrol (XAN) in $CDCl_3$

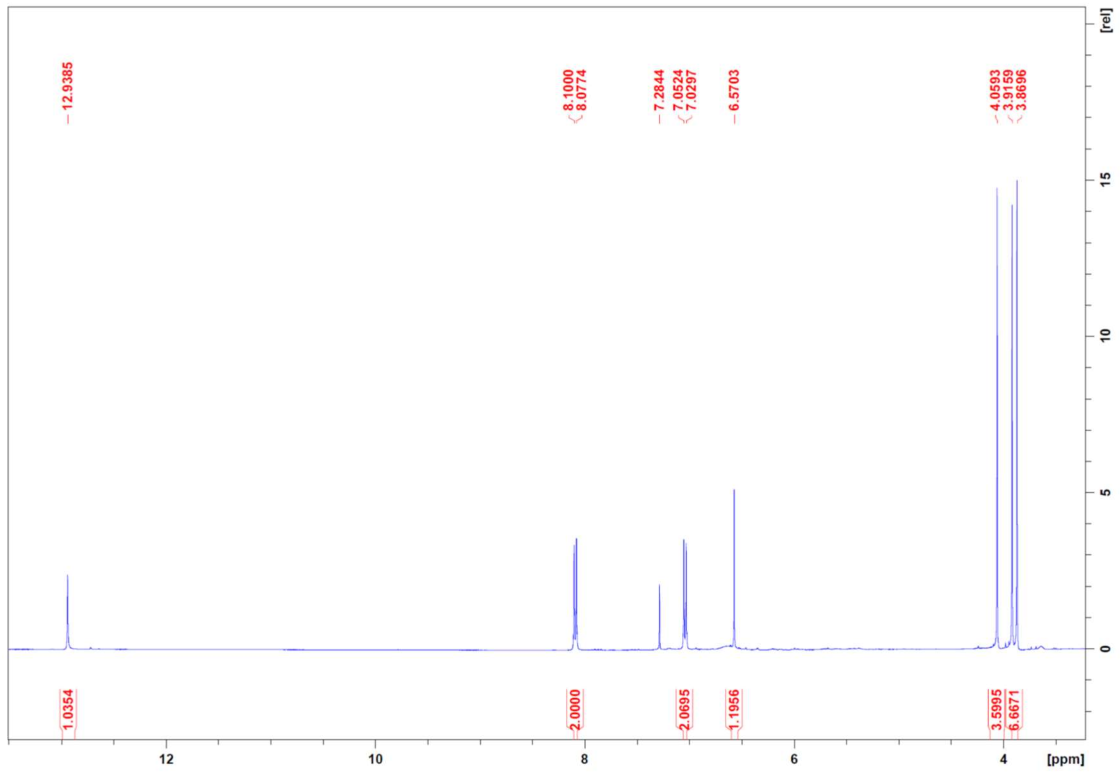

Figure S1. ^{1}H NMR (400 MHz) of xanthomicrol (XAN) in $CDCl_3$.
